# An inexpensive UV-LED photoacoustic based real-time sensor-system detecting exhaled trace-acetone

**DOI:** 10.1016/j.pacs.2024.100604

**Published:** 2024-03-21

**Authors:** Jonas Pangerl, Pritam Sukul, Thomas Rück, Patricia Fuchs, Stefan Weigl, Wolfram Miekisch, Rudolf Bierl, Frank-Michael Matysik

**Affiliations:** a Sensorik-ApplikationsZentrum (SappZ), Regensburg University of Applied Sciences, Regensburg 93053, Germany; b Institute of Analytical Chemistry, Chemo- and Biosensing, University of Regensburg, Regensburg 93053, Germany; c Rostock Medical Breath Analytics and Technologies (RoMBAT), Dept. of Anaesthesiology, Intensive Care Medicine and Pain Therapy, University Medicine Rostock, Rostock 18057, Germany

**Keywords:** Photoacoustic spectroscopy, Real-time mass-spectrometry, Breath analysis, Acetone, UV-LED

## Abstract

In this research we present a low-cost system for breath acetone analysis based on UV-LED photoacoustic spectroscopy. We considered the end-tidal phase of exhalation, which represents the systemic concentrations of volatile organic compounds (VOCs) – providing clinically relevant information about the human health. This is achieved via the development of a CO_2_-triggered breath sampling system, which collected alveolar breath over several minutes in sterile and inert containers. A real-time mass spectrometer is coupled to serve as a reference device for calibration measurements and subsequent breath analysis. The new sensor system provided a 3σ detection limit of 8.3 ppbV and an NNEA of 1.4E-9 Wcm^−1^Hz^−0.5^. In terms of the performed breath analysis measurements, 12 out of 13 fell within the error margin of the photoacoustic measurement system, demonstrating the reliability of the measurements in the field.

## 1 Introduction

The utilization of human breath analysis as a diagnostic tool presents a significant challenge. For instance, more than 870 VOCs have been discovered in exhaled breath [1]. Some of them serve as biomarkers, which are typically categorized as endogenous compounds that either emanate from regular metabolic processes or reveal an imbalance, potentially indicating disease or malfunction within the body or organs. Given the non-invasive, uncomplicated, and painless nature of breath sampling, breath analysis finds utility in various domains of clinical medicine [2], [3], [4]. Human exhaled breath predominantly comprises nitrogen (74%V), oxygen (14–16%V), carbon dioxide (up to 5%V), water vapor (2.9–5%V), and argon (1%V) [5], [6], [7]. However, only a tiny fraction of exhaled breath contains VOCs that carry physio-metabolically and clinically relevant information [8]. The specific fraction varies depending on the subject’s age, health, and individual metabolic profile [9]. In this context, the volume fraction of different biomarkers ranges from parts per trillion by volume (pptV) to lower parts per million by volume (ppmV) [10]. Nevertheless, a correct quantification of these low concentrations of biomarkers is the basis for generating dependable diagnostic data.

Photoacoustic spectroscopy (PAS) represents a promising approach to address these demands. Notably, its attributes of high sensitivity, spectral selectivity, and potential for miniaturization render PAS well-suited for breath analysis as reviewed in [10]. The increasing popularity of PAS in recent years is attributable to its utilization of cost-effective components, such as 3D-printed measurement cells and cell phone microphones [11], [12], [13]. In view of photoacoustic breath monitoring in general, Popa et al. studied the impact of wearing surgical face masks on the concentrations of the exhaled respiratory gas components CO_2_ and ethylene [14]. For basic and applied breathomics (e.g., of unidentified VOC marker profiles) established gold standards like Gas-Chromatography Mass-Spectrometry (GC-MS) and real-time Proton-Transfer-Reaction Time-of-Flight Mass-Spectrometry (PTR-TOF-MS) represent the state of the art. As soon as certain markers are well-established as for breath isoprene [15] or acetone, PAS offers a compelling evidence of performance to comprehend potential pre-clinical point-of-care applications.

Acetone (C_3_H_6_O) belongs to the ketones and is a well-known VOC in human breath with a relatively high abundance that ranges normally between 0.2 and 0.9 ppmV among heathy adults [16]. It is formed at cellular and organ levels as a byproduct of glycolysis and/or lipolysis and is transported to the lungs via the bloodstream, where it is exhaled through the alveolar blood-gas interface. The monitoring of acetone is of interest in certain applications, including its use in assessing the anaerobic threshold of athletes to optimize performance [17], [18]. However, it is also considered a potential biomarker for type 1 and type 2 diabetes mellitus, although a definitive and consistent association has not been established yet [19]. While studies have shown a linear relationship between breath acetone levels and blood glucose concentrations [20], [21], acetone levels exhibit significant variations, making it unsuitable as a reasonable substitute for traditional blood glucose testing methods [19]. Nevertheless, acetone is recognized as a biomarker for ketogenic diets. Monitoring acetone levels can aid in weight loss efforts [22], [23], [24] and provide support to children with epilepsy following a ketogenic diet [25], [26]. Furthermore, acetone’s clinical relevance as a biomarker has been highlighted in studies by Samara et al., which reported elevated levels of acetone and pentane in patients with acute decompensated heart failure (ADHF) [27]. In view of photoacoustic acetone detection, Bratu et al. and Tyas et al. exploited a CO_2_ laser for explosives detection and diabetes mellitus patients monitoring with a detection limit of 30 ppbV, respectively [28], [29]. Moreover, Kidavu et al. applied an UV laser, a Mid-IR QCL and a THz source yielding acetone detection limits of 5.05 ppbV, 7.92 ppbV and 15.3 pptV using a Helmholtz photoacoustic cell, respectively [30]. Our group recently achieved a sub-ppbV detection limit of 0.25 ppbV by applying a lock-in time constant of 10 s at laboratory conditions with a QCL-PAS setup at 8.26 µm [31]. As a cost-effective alternative, Weigl et al. developed a measurement system based on a modulated UV LED with a limit of detection (LoD) (3σ) of 20 ppbV under controlled laboratory conditions [12]. In this work, the approach of Weigl et al. is continued and elaborated. Thus, the acetone content of alveolar breath could indeed be determined by an UV-LED photoacoustic setup. Due to the effectiveness of UV-C light as a tool for water disinfection [32] and against respiratory viruses such as SARS-CoV-2 [33], the COVID-19 pandemic has accelerated the development of powerful yet cost-effective UV-LEDs.

To the best of our knowledge, the first setup of UV- PAS for detecting acetone was reported by Oka et al. in 1988 [34]. They exploited the broad acetone absorption band centered around 278 nm, arising from the transition of an oxygen n_S_-orbital electron to the excited $\pi_{s}^{*}$-orbital (n_S_ → $\pi_{s}^{*}$). In their study, they employed a 500 W xenon arc lamp emitting light within the range between 230 – 340 nm. Subsequently, in 2015, Preukschat et al. conducted a comparative analysis of the LoDs for acetone detection using an Optical Parametric Oscillator (OPO) to excite vibrational and electronic states in the Infrared (IR) region and with a 266 nm UV laser, respectively[35].

In conclusion, PAS has demonstrated its applicability of trace gas detection, exploiting IR and UV spectral regions. As acetone serves as an interesting and valuable biomarker, this work presents an UV LED-based photoacoustic breath acetone sensor, primarily focused on probe sampling of alveolar breath and the detection of acetone towards a potential application in modern breath analysis. The novelty of this research lies in probe sampling of the end-tidal breath as well as the deployment of an inexpensive UV-LED as the light source, emitting at the peak of the acetone absorption band (σ_acetone_ = 5.06E-20 cm^2^) around 278 nm. The design of the photoacoustic measurement cell aimed a high-performing, compact sensor, thereby facilitating future integration into a compact device.

## 2 Theory

PAS is based on the photoacoustic effect, initially observed by Bell in 1880 [36]. This technique, which is based on the principles of classical absorption spectroscopy, uses an amplitude-modulated light source to excite molecules. During the LED’s off-phase, excited molecules relax, finally causing a periodic heat input, resulting in pressure fluctuations, i.e. an acoustic signal. To enhance this signal, the LED modulation frequency is matched with the acoustic resonance frequency of the photoacoustic cell. The resulting photoacoustic signal delivered from a Lock-In-Amplifier (LIA) $U_{\text{LIA}}$ corresponds to the photoacoustic pressure $p_{a}\left({\overset{⃑}{r}}_{\mathrm{mic}}\right)$ in the first longitudinal mode of the resonator at microphone position ${\overset{⃑}{r}}_{\mathrm{mic}}$.

(1) $$\begin{array}{lll} U_{\mathrm{LIA}} & = & \frac{1}{\sqrt{2}}\cdot U_{\mathrm{mic}}=\\ & = & C_{\mathrm{cor}}\;B_{\mathrm{mic}}\underset{p_{a}\left({\overset{¯}{r}}_{\mathrm{mic}}\right)}{\underset{⏟}{\left(\gamma -1\right)\frac{Q}{2\pi f_{\mathrm{res}}}\frac{L_{R}}{V_{R}}\frac{N_{A}}{V_{\mathrm{mol}}}N_{i}\sigma_{i}\left({\overset{∼}{v}}_{\mathrm{Ph}}\right)P_{0}\left({\overset{∼}{v}}_{\mathrm{Ph}}\right)\in_{\mathrm{relax}}}} \end{array}$$

Thus, besides the analyte concentration represented by the volume ratio $N_{i}$, the photoacoustic pressure is also proportional to the decremented heat capacity ratio $\left(\gamma -1\right)$, the ratio of the resonator’s quality factor and its resonance frequency $\frac{Q}{{2\pi f}_{\mathrm{res}}}$, the ratio of effective resonator length and volume $\frac{L_{R}}{V_{R}}$, as well as to the absorption cross-section of the analyte at the emission wavenumber $\sigma ({\tilde{\nu }}_{\mathrm{Ph}})$. Furthermore, it is proportional to the respective optical power of the light source $P_{0}({\tilde{\nu }}_{\mathrm{Ph}})$, and the relaxation efficiency $\epsilon_{\text{relax}}$. The molar volume ${\text{V}}_{\text{mol}}=\frac{\text{R}T}{p}$, where R is the ideal gas constant, affects the photoacoustic signal by $p_{a}∼\frac{p}{T}$. The converted voltage signal further depends on the microphone sensitivity ${\text{B}}_{\text{mic}}$ and an empirically determined refinement factor ${\text{C}}_{\text{cor}}$ compensating for different cell-specific constants summarized in [37]. The heat capacity ratio γ of a gas matrix that contains n compontes $\sum_{i=0}^{n}N_{i}=1$ was calculated after

(2) $$\begin{array}{c} \gamma =\sum_{i=0}^{n}N_{i}\frac{C_{p,i}R}{C_{p,i}R-R}\;\\ \mathrm{with}\;C_{p,i}\approx a_{1i}+a_{2i}T+a_{3i}T^{2}+a_{4i}T^{3}+a_{5i}T^{4} \end{array}$$

where $C_{p}$ is the isobar heat capacity and T the temperature of the sample. The approximation for the $C_{p}$ values as well as the polynomic constants $a_{m}$ ($m\in \left[1;5\right]$) for all components i of the sample gas matrices are adapted from Burcat et al. [38]. For a more extensive derivation of the photoacoustic pressure, the interested reader is referred to [11], [39], [40].

However, the excitation of acetone in the UV range at approx. 278 nm, i.e. 35971 cm^−1^ or approx. 4.46 eV, can induce photodissociation or photolysis processes. These are initiated by the absorption of the UV photons, which results in the transition of the acetone molecule to a higher electronic state. By transiting to a higher state or during relaxation back to ground state higher vibrational states are excited, which can lead to the breaking of chemical bonds, particularly C-C and C-H bonds, within the acetone molecule. Consequently, the acetone molecule may decompose into fragments, e.g. the acetyl radical (CH_3_CO), hydrogen (H), or carbon monoxide (CO). The specific pathways of fragmentation depend not only on the energy of photon absorption but also on the prevailing pressure, temperature, and bulk matrix composition. Fourier-Transform-InfraRed spectroscopy (FTIR) measurements performed by Weigl et al. have revealed that, at an acetone concentration of 48.55 ppmV and a flow rate of 300 ml/min illuminated by a UV LED emitting at 278 nm with 88 mW optical power, only 5‰ of the acetone molecules were dissociated [12]. At realistic breath gas concentrations around 500 ppbV, this amounts to only 2.5 ppbV, which is negligible in terms of clinically relevant findings and therefore not considered within the experiments of this work. Readers seeking a more in-depth exploration of this phenomenon are encouraged to refer to related literature [35], [41], [42].

## 3 Materials and methods

**Fig. 1:**
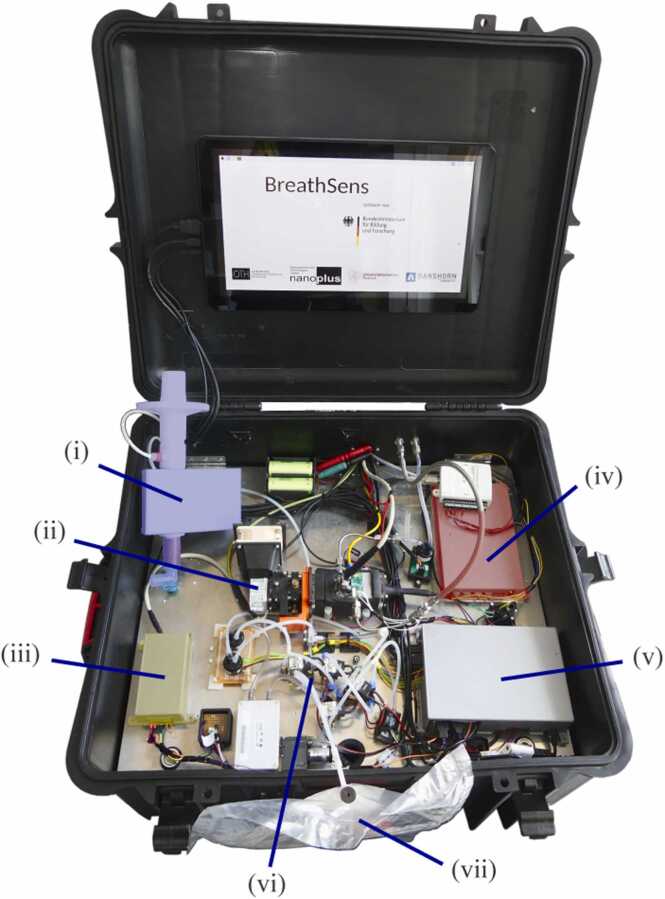
Breath acetone sensor system integrated into a robust case. Power supplies as well as the TEC driver for temperature control are mounted on the bottom side of the metal plate. (i) – Mouthpiece (SpiroScout); (ii) – photoacoustic sensor element; (iii) – LED driver; (iv) – LIA; (v) – RaspberryPi; (vi) – pneumatic components (valves, sensors, and pumps) of the probe collecting system; (vii) – TB.

This section presents the underlying components and procedures for reliable breath measurements in detail since no comparable system has been reported so far. In short, this includes the probe sampling and supply to the sensor system, a description of the low-cost photoacoustic sensor and the concept of data evaluation that has been applied. To create a portable and robust demonstrator setup, all components of the breath acetone sensor were integrated into a robust case (see Fig. 1). Therein, an intermediate metal plate was integrated, with different switch-mode power supplies (from Meanwell Enterprises Co., LTD., Taiwan) and the Thermoelectric Cooler (TEC) driver (TEC-1091, Meerstetter Engineering GmbH, Switzerland) for temperature control of the sensing element being attached to the bottom side. The measurement electronics were placed above the metal plate. Those consisted of an LED driver from Lasertack (iLD-2500, Lasertack GmbH, Germany) (iii) a printed circuit board (PCB) LIA from Femto (LIA-BVD-150-L, Femto Messtechnik GmbH, Germany) (iv) and a RaspberryPi (v), with a screen mounted on the inside of the case lid. The RaspberryPi is further extended by a frequency generator (FG) chip (AD9833) providing the modulation and reference frequency for the LED driver and the LIA reference input, respectively. The microphone (ICS-40720, InvenSense Inc., US) with a sensitivity of 25.1 mV/Pa was powered by two very voltage stable Nonophosphate Lithium-Ion batteries. Electromagnetic Compatibility (EMC) was ensured by shielded housing of PCB electronics and the ground connected intermediate metal plate. The sampling system implies a mouthpiece (i), the pneumatic components (vi) and a TB (vii), where breath samples are collected.

### 3.1 Breath sampling

**Fig. 2:**
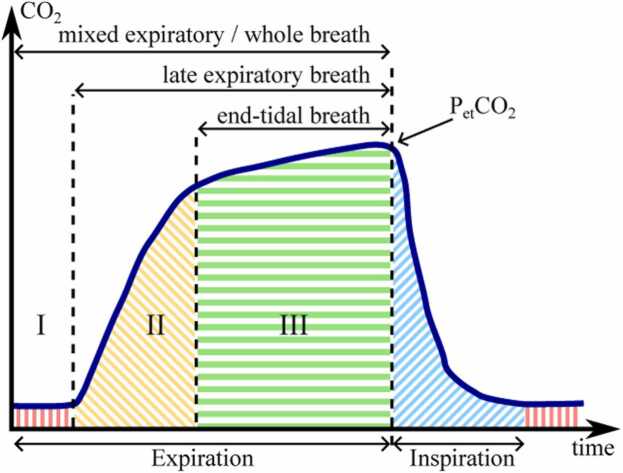
Capnogram of a respiratory cycle during breath measurements visualizing the exhalation phases I, II, and III. The schematic is adapted from [44].

Breath sampling can be controlled by monitoring the human respiratory cycle via capnography, which displays exhaled CO_2_ as a marker for gas exchange in real-time. During breathing, the CO_2_ content changes periodically as illustrated by the capnogram in Fig. 2. Accordingly, breath samples can be classified into three portions [43]: *Mixed expiratory breath / whole exhalation*, which comprises dead space from the upper-airways (Phase I), trachea and lower-airways (Phase II) and alveolar air (Phase III). This breath portion is susceptible to environmental contaminants, rendering it less suitable for precise VOC breath analysis. *Late expiratory breath* includes fractions from Phases II and III. The most accurate method for collecting alveolar air is to only take *end-tidal breath* (Phase III) initiating when the capnogram flattens and attains the partial-pressure of end-tidal CO_2_ (P_et_CO_2_). This portion of exhaled breath represents the systemic concentrations of exhaled VOCs and is the basis for the breath measurements of this research.

Exhaled VOC concentrations are influenced by various factors, including lifestyle, physiology, and health. Changes can occur in seconds by alternating breathing patterns [45], [46], minutes (due to posture changes [47]), hours (after meals [48]), and over longer periods (menstrual cycles [49], pregnancy [50], and aging [51], [52]). Research has shifted its focus to understanding these factors affecting VOC concentrations in exhaled breath, recognizing the crucial influence of our physiology and metabolism [9]. To mitigate the impact of physio-metabolic effects, standardizing breath sampling procedures is essential, guided by the efforts of organizations like the *International Association of Breath Research (IABR)* [53], [54], which are summarized in [9] and further explained in the literature cited therein. These aspects were considered during the sampling process of this research. For collecting the breath sample, so-called Tedlar® Bags (TB) made of polyvinyl fluoride were used. These breath containers are commonly used, inexpensive, inert and can be used multiple times [55]. Since fast diffusion of polar VOCs like acetone through the bag wall has been reported in literature [56], storage of samples was avoided, and they were analyzed immediately after collection.

**Fig. 3:**
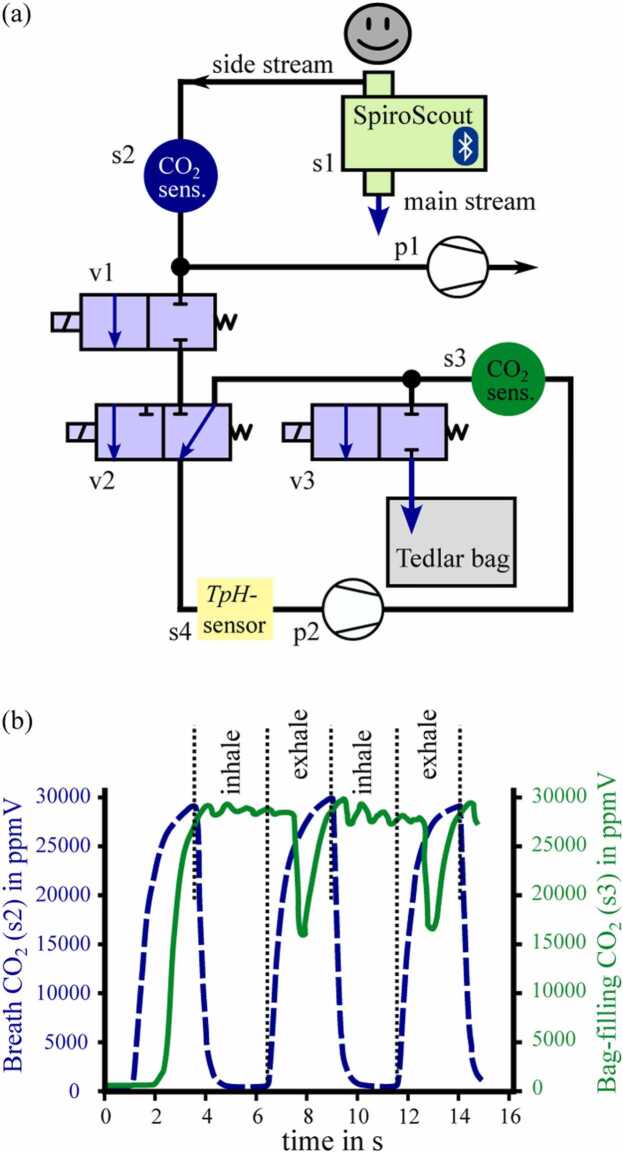
Schematic of the probe extraction system (a) and CO_2_ levels during breathing and bag-filling (b). s1 – SpiroScout; s2, s3 – MDIR-CO_2_-Sensors; s4 – TpH-Sensor; p1, p2 – diaphragm pumps; v1-v3 – magnet valves.

Since only the exhaled air at the alveolar plateau (end-tidal breath) correlates well to blood concentrations of VOCs [43], only this region of the exhalation cycle (see Fig. 2) was investigated. Therefore, a CO_2_ level triggered system was developed as illustrated in Fig. 3a. As the concentration of exhaled VOCs depends on many extrinsic and intrinsic factors [57], [58], [59], a standardization for sampling is targeted in modern breath analysis [9]. For instance, Sukul et al. explicitly showed that breathing against a resistance changes the composition of VOC profiles [60]. For this reason, the SpiroScout (s1) from Ganshorn (Ganshorn Medizin Electronic GmbH, Niederlauer, Germany), an ultrasound-based spirometer for pulmonary function diagnostics, was used as the central element for breath probe sampling, which allows aspiration of breath through a side-stream connection providing no flow resistance during breathing. Once sampling is started, pump p1 draws the sample through the side-stream. If the CO_2_ concentration in sensor s2 exceeds the threshold set at 2.5%V-CO_2_, the valves v1, v2 and v3 switch, pump p1 stops and the pump p2 starts to collect the alveolar breath sample into the TB. The empirically determined optimal flow rates of the pumps are 800 ml/min for p1 and 100 ml/min for p2, respectively. If the CO_2_ concentration in s2 is below 2.5%V-CO_2_, i.e. whilst inhalation, the valves reset to their initial position and pump p1 resumes operation. Thus, the relevant exhalation phase of many breathing cycles can be stored in the TB with a capacity of up to 1.5 liters. During the experiments, patients breathed through the SpiroScout for approximately 6 minutes until the TB was about half full. This sample volume was needed to perform reliable photoacoustic measurements. The CO_2_ sensor values s2 and s3 are plotted against time in Fig. 3b. The s2 values represented by the dashed dark blue graph show the breathing cycles with their different phases according to Fig. 2, the s3 values visualized in green replicated the CO_2_ value of the probes stored in the TB. The peaks observed within the s3 values are assumed to result from valve switching, wherein a small amount of ambient air present in the dead space of the piping during the inhalation process between the junction point to s2 and v1 is drawn into the circuit. The oscillation of the CO_2_ level is a result of the low flow rate of the diaphragm pump p2. Spirometry data and communication of the SpiroScout are transmitted via Bluetooth, the pumps and valves are connected to the GPIO and the CO_2_ sensors as well as the Temperature-pressure-Humidity (TpH) sensor (BME680, Bosch Sensoric GmbH, Germany) were connected to the UART outputs of the RaspberryPi microcontroller, respectively.

Initially, we tried to perform the photoacoustic breath acetone measurement in real time, i.e. to integrate the photoacoustic measuring cell serially into the probe gas flow after p2. Unfortunately, the sample volume was too small and oscillations in mass flow due to the diaphragm pumps and the magnetic valves influenced the microphone signal as well as the LIA significantly, rendering the resulting signal unusable. For this reason, we proceeded with the two-step and physically separated procedure presented here, i.e. a sufficient sample volume is collected before the analysis is performed in a second step. Four major advantages of this strategy are the following:

(i) Only the clinically relevant breath phase is stored over many cycles. This averaging of VOCs significantly reduces the standard deviation between samples.
(ii) A constant mass flow can be maintained over a longer period of time with consistent sample composition. Thus, the photoacoustic data can be averaged, establishing near-laboratory conditions in real-time.
(iii) There is no electromagnetic crosstalk or vibrations from the valves or pumps affecting the electronics of the sensor.
(iv) UV photodissociation from acetone due to continuously pumping the sample through the cyclic measurement system does not have to be considered.

Therefore, the design of the probe extraction system as a cycle by means of v2 is in fact not necessary and a relic from real-time measurement tests. The following sections describe the procedures of reference sampling and supplying sample gas to the sensor as well as a description of the photoacoustic sensor system.

### 3.2 Reference sampling and sample supply to the sensor

**Fig. 4:**
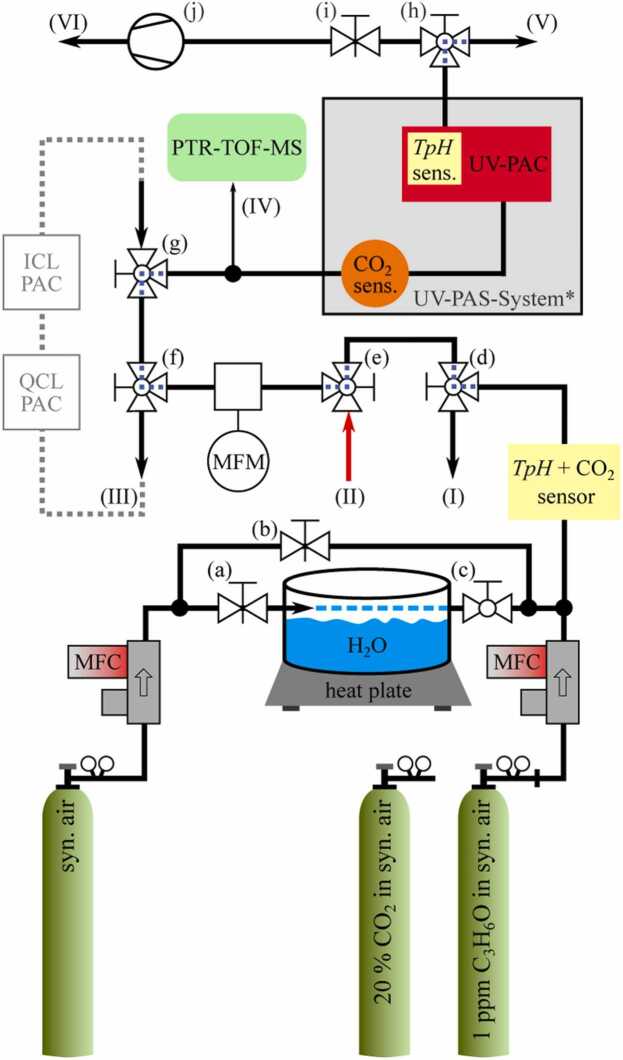
Gas stream for calibration and breath measurements: (I) – outlet for bag filling, (II) – Breath probe inlet, (III) – QCL/ICL loop, (IV) – PTR transfer line, (V) – exhaust during MFC mode, (VI) – exhaust during bag mode; (a) and (b) – valves for adjusting humidity; (c) – Humidity switch-off; (d), (e) and (h) – Switching between MFC and bag mode; (f) and (g) – connect/shortcut ICL and QCL system; (i) – adjustment of the flow during bag mode. The dotted line within valves (d)-(h) indicate the default state, if those valves are switched, the open end is connected, i.e. the connector rotates by 90 degrees. *The UV-PAS-System is further depicted within Fig. 1.

Besides this breath sample extraction system, a versatile gas distribution system was developed to alternatively fill Tedlar Bags with synthetic breath samples and to supply photoacoustic sensors as well as the reference system either with samples from Tedlar Bags or directly with reference gas from cylinders. This gas distribution system was mounted on a trolley for mobile measurements. A schematic of this system is provided in Fig. 4. The different valving positions of valves (d), (e) and (h) allow to change between different modes summarized in Table 1. These are a *Bag-Fill mode* to fill Tedlar Bags with reference gas from cylinders (synthetic breath samples), a *Bag mode* to supply the sensors with samples stored in bags and a *MFC mode* to supply the sensors with reference gas from directly from cylinders. The schematic of the gas distribution system in Fig. 4 additionally shows two valves (f) and (g) (connection III) to integrate further photoacoustic sensor systems to the gas stream, which however, won’t be further addressed in this work.

**Table 1:** Modes due to different configurations of the valve switches: According to Fig. 4, 0 indicates default valve position, 1 a switched valve, i.e., a rotation of the dashed angle by 90 degrees, X implies an irrelevant valve position.

Mode	(d)	(e)	(h)	description
MFC	0	0	0	Operation via gas tanks / laboratory setup
Bag	X	1	1	Operation via TB and vacuum pump (j)
Bag-Fill	1	X	X	Filling of TB by the gas tanks

The total mass flow within this gas distribution system was monitored by a mass flow meter (MFM) (M-500SCCM, Alicat Scientific, US). A 3D printed T-connector sealed by an epoxy resin was placed upstream of the UV-PAS system, serving as an adapter towards a real-time mass spectrometer, i.e., a PTR-TOF-MS (PTR-TOF 1000, Ionicon Analytik GmbH, Austria). This reference instrument connected to port (IV) actively draws in the sample at a rate of 20 ml/min. To verify the end-tidal-breath of a sample and to correctly consider the composition of the bulk matrix in terms of signal evaluation, an MDIR CO_2_ sensor sampling with 5 Hz (SPrintIR-WF-20, Gas Sensing Solutions LDT, UK) was integrated into the UV-PAS system. The corresponding humidity value of the bulk matrix is provided by the TpH sensor directly integrated into the photoacoustic measuring cell.

#### 3.2.1 MFC mode

The MFC mode is similar to a laboratory setup as described in [11]. Applying MFC mode, prediluted analyte can be further diluted using mass flow controllers (MFC) (F-201CV-500-XXX, Bronkhorst GmbH, Germany). To humidify samples, a humidification system has been integrated which is already described in [61]. Therefore, the humidity in the gas flow is adjusted via the needle valves (a) and (b). A shut-off valve (c) is provided for rapid deactivating of humidification. Since the analyte acetone would dissolve in water when streaming over the water surface of the tank, only non-analyte dilution gas was humidified. Thus, the maximum humidity was limited, depending on the mass flow rates through (a) and (b). However, in order to mimic realistic breath conditions, the aluminium humidity tank was heated to approximately 35 °C. By also heating the pipes downstream of the tank, a higher absolute humidity could be achieved in the sample gas without condensation. This system allows to set humidities of up to 3.5%V-H_2_O and a typical end-tidal CO_2_ concentration of 4%V. A combination of TpH sensor and CO_2_ sensor was integrated to monitor CO_2_ and water as well as pressure and temperature within the gas stream. These variables are particularly important when filling TB in terms of reference measurements (Bag-Fill mode). Downstream the UV-PAS system, the sample is released to the room air via output (V). When MFC mode was applied, the mass flow was set to 500 ml/min, an empirical value that allows both, fast flushing and a quick change of analyte concentration or humidity. Westfalen AG (Münster, Germany) provided the gases for all experiments. The purity of the dilution gas synthetic air (SA) was specified with less than 0.1 ppmV of hydrocarbons. The analyte gas acetone was diluted in SA and specified with a concentration of 1.0 ppmV and an accuracy of 10% and the bulk component CO_2_ was specified by a concentration of 20%V diluted in SA with a 2% accuracy, respectively.

#### 3.2.2 Bag mode

In Bag mode, TB can be analysed either filled with real breath samples as described in Section 3.1 or with synthetic breath samples via the bag-fill mode. Therefore, a TB is connected to input (II). The mass flow of the TB can be controlled by the MFM and needle valve (i) since this valve serves as the pre-pressure regulator of the applied miniaturized vacuum pump (j). After the photoacoustic cell (PAC), the sample gas is released via outlet (VI) downstream of the pump. In contrast to measurements in MFC mode, measurements in Bag mode are performed at negative pressure (around 900–950 mbar) using pump (j) and valve (i). The absolute pressure difference between MFC mode and Bag mode is between 50 and 100 mbar, depending on the set mass flow. To achieve longer periods of constant flow and thus more accurate photoacoustic measurements, the flow rate of bag samples was set between 200 and 300 ml/min. Below a rate of 150 ml/min the pressure within the measuring cell decreases due to the high vacuum pressure gradient towards the pump, significantly affecting the *Q*-factor and reducing the number of molecules contributing to photoacoustic sound wave generation and transmission.

#### 3.2.3 Bag-Fill mode

When using the Bag-Fill mode, an empty TB is connected to output (I). A synthetic breath sample can be filled via the gas mixing and humidification system. The sample can then be analysed in Bag mode afterwards. The Bag-Fill mode can be used for reference background signal measurements without analyte. In terms of these measurements, the bulk matrix is set as similar as possible to a previous breath measurement. The discrepancy of the breath and reference sensor readings then results from UV absorption by breath acetone. Before a synthetic or real breath sample is collected within a TB, the bags are cleaned twice by flushing with SA and subsequent vacuuming.

### 3.3 Low-cost photoacoustic acetone sensor

**Fig. 5:**
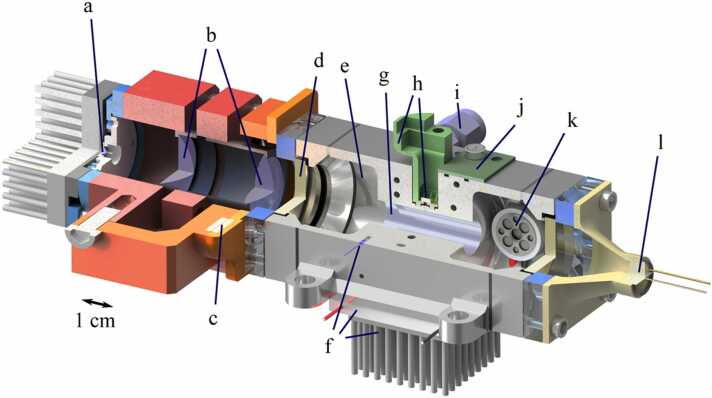
True-to-scale render drawing in three-quarter section of the photoacoustic measuring cell: a – UV-LED with hemispherical lens mounted on a heat sink, b – Optics (aspheric and biconvex lens), c – magnet flange, d – windows, e – buffer volume, f – temperature control circuit (NTC, Peltier-element, heat sink), g – resonator, h – microphone and 3d printed holder, i – gas inlet, j – TpH sensor, k – speaker, l – photo diode.

The core of the UV breath acetone analyzer is an integrated photoacoustic measuring cell, which is depicted as a true-to-scale rendering in three-quarter section in Fig. 5. This system primarily employs low-cost components involving inexpensive housing components, in-house 3D printing, and the use of cost-effective mass-produced parts showcasing remarkable affordability for an optical system. The components applied to the measuring cell are listed in Table 2.

**Table 2:** Part list with designation, and manufacturer of the components assembled within the PAS Acetone sensor. The letters in the first column correspond to the labelling according to Fig. 5.

Position	Description	Specification	Manufacturer
(a)	UV-LED	LB686-UVC-275 nm-D4	Ivy Bridge Technology (IBT), China
(b)-1	Aspherical lens	No. 33955	Edmund Optics, US
(b)-2	Biconvex lens	No. 46290	Edmund Optics, US
(c)	Magnets	QA-10×10×2-NI-N52	MTS Trading UG, Germany
(d)	Windows	No. 65875	Edmund Optics, US
(f)-1	NTC	B57861S	TDK Electronics, Germany
(f)-2	Peltier-Element	ET-161–12–08-E	European Thermodynamics Ltd,
(h)	Microphone	ICS-40720	TDK InvenSense, Germany
(i)	Gas Stream Connector	SS-200–1-M5×0.8RS	Swagelok, US
(j)	TpH-Sensor	BME680	Bosch Sensoric GmbH, Germany
(k)	Speaker	K16	Visation GmbH, Germany
(l)	Photodiode	TOCON_ABC8	Sglux SolGel Technologies GmbH, Germany

The UV acetone sensor can basically be divided into two parts, i.e., the optical system and the photoacoustic cell which is connected to the gas flow. By means of a magnetic flange and dowel pins, the optics can be separated from the measuring cell in a modular way, allowing to connect our standard measuring cell (see [11], [12], [31]) to the light source in a simple and reproducible way. The UV-LED (a) already incorporates a hemispherical lens to reduce the numerical aperture. The additional optical components (b) comprise an aspherical lens and a biconvex lens. The selection of these lenses has been specified in [12]. The PAC features large 1” windows (d) as well as a 1 cm diameter resonator (g) to focus a maximum of the emitted light to the resonator center. To monitor degradation processes of the UV-LED, a UV photodiode (l) is mounted to the out-coupling window fixed by a 3D printed holder. The temperature of the gas measuring cell is controlled to 39 °C to prevent condensation of breath humidity by a temperature control circuit (f). The acoustic resonator tube is 5 mm in radius and 31 mm in length. For further details about geometry and design of the PAC, the interested reader is referred to a previous publication [12]. The actual sound transducer, i.e., the MEMS microphone, is placed above a 1 mm diameter outcoupling hole in the middle of the resonator and is fixed by means of a 3D printed holder (h). Due to different bulk compositions and the limited amount of sample volume, the resonance frequency of the sample has to be determined rapidly for each breath sample. Therefore, an Acoustic Resonance Monitoring System (ARMS) involving a speaker (k) was integrated as described in [37]. The sample enters the measurement chamber via Swagelok connections (i) and a preheating channel incorporated to the PAC monitored by a TpH sensor on an in-house developed PCB (j).

In contrast to laser sources, LEDs suffer from disproportionate decrease of optical power and reflections along the optical path within the PAC. The typical continuous wave (cw) radiant power of 290 mW specified in the data sheet is bisected to 145 mW due to modulation with a 50% duty-cycle. However, only about 16 mW of this could be measured directly after the LED with a PM100D power meter which was equipped with a S302C thermal detector (Thorlabs GmbH, Germany) featuring a diameter of 10 mm and placed 15 mm behind the inlet of the power meter. Through the use of optics, a major part of the beam could be focused, thus increasing the measured optical power to almost 60 mW after an optical path length of 10.4 cm, which equals 41% of the emission power. Behind the measurement cell, due to absorption losses at the windows and reflection losses at the cell walls, a power of approx. 21 mW remained. In fact, the actual optical power contributing to the photoacoustic signal is higher due to reflections form the resonator walls.

### 3.4 Data evaluation

Since all breath samples were collected with sufficient sample volume, measurements of approximately three minutes were carried out with a mass flow rate of approximately 300 ml/min. Within this period, three individual measurements resulting from averaging 100 data points with a sampling rate of 5 Hz were performed. These three individual measurements were again averaged finally yielding the concentration reading and accuracy of the sensor within a total of 60 seconds measuring time. The roll-off and integration time of the LIA have been set to 12 dB/octave and 3 seconds, respectively. Since an LED is used as an emitter, the signal-to-background-signal ratio (SBSR) is significantly lower (approx. 8% at 600 ppbV acetone) than in conventional laser-PAS measurements due to poor beam quality and, hence, increased light absorption by the cell walls. As a consequence of the pressure dependency of the background signal, the raw data were further pressure normalized.

(3) $$U_{\mathrm{LIA},\mathrm{norm}}=U_{\mathrm{LIA}}+U_{\mathrm{LIA}}\left(p_{\mathrm{ref}}-p\right)b_{p}$$

In (2), $U_{\mathrm{LIA},\mathrm{norm}}$ is the pressure normalized photoacoustic magnitude, $p_{\mathrm{ref}}$ the reference pressure, which was set to the ambient pressure at sea level (1013.25 mbar), p the pressure within the measuring cell, and $b_{p}$ the coefficient of pressure induced signal increase, which was empirically determined to 6.3E-4 mbar^−1^. As a next step, the measurement signal was offset-corrected by subtracting the absolute value of the pressure normalized background amplitude $U_{\mathrm{LIA},\mathrm{BS},\mathrm{norm}}$.^1^ Following each breath measurement in bag-mode, a background signal measurement was carried out in MFC-mode, where the bulk composition was adjusted to approximately match the real breath sample, excluding acetone. The resulting difference after offset correction represents the photoacoustic amplitude $U_{\mathrm{PAS},\mathrm{norm}}$, which is used to calculate the acetone concentration within the sample.

(4) $$U_{\mathrm{PAS},\mathrm{norm}}=U_{\mathrm{LIA},\mathrm{norm}}-U_{\mathrm{LIA},\mathrm{BS},\mathrm{norm}}$$

The influence of bulk composition, in particular the volume fractions of CO_2_ and water, shows no spectral effects on the UV acetone PAS measurements. The effects on the acoustic parameters, i.e., quality factor and frequency of acoustic resonance, are determined individually by the ARMS. Knowing the bulk composition of the sample and temperature, influences on the heat capacity ratio can be calculated via Eq. 2. By additionally monitoring pressure and optical power, all necessary quantities for calculating the acetone concentration are determined and thus, the analyte volume ratio can finally be calculated by solving Eq. 1 for $N_{i}$ and substituting $U_{\mathrm{LIA}}$ by $U_{\mathrm{PAS},\mathrm{norm}}$:

(5) $$N_{i}=U_{\mathrm{PAS},\mathrm{norm}}\frac{2\pi f_{\text{res}}V_{R}V_{\text{mol}}}{{\text{C}}_{\text{cor}}{\text{B}}_{\text{mic}}\left(\gamma -1\right)Q\;L_{R}{\text{N}}_{\text{A}}\sigma_{i}\left({\tilde{\nu }}_{\mathrm{Ph}}\right)P_{0}\left({\tilde{\nu }}_{\mathrm{Ph}}\right)\epsilon_{\text{relax}}}$$

Table 3 summarizes all values used for this concentration conversion. The refinement factor encompasses various phenomena including the efficiency of acoustic coupling between the resonator and microphone, the light-to-sound coupling, and minor deviations in the measurement of optical power attributable to the absorption of the windows [37]. However, due to the divergent emission of light from an LED, it becomes unfeasible to directly measure the true optical power contributing to the generation of the photoacoustic signal. The installed photodiode serves merely as a reference for monitoring LED degradation processes. It is therefore assumed that the actual optical power contributing to the photoacoustic signal is at least a factor of ${\text{C}}_{\text{cor}}$ higher and is thus in the region of approx. 100 mW. As no relaxation loss phenomena have been identified, the efficiency of relaxation $\epsilon_{\text{relax}}$ is assumed to equal 1.

**Table 3:** Explanation of the individual elements of Eq. 4. “var” indicates a variable value for each measurement and “n.d.” a not defined value, respectively.

Symbol	Unit	Value	Source
$U_{\mathrm{LIA},\mathrm{norm}}$	V	*var*	Measured and compensated PA magnitude
$C_{\mathrm{cor}}$	-	4.834	Determined upon sensor calibration
$B_{\mathrm{mic}}$	V/Pa	0.0251	Datasheet value (-32 dBV)
γ	-	*var*	Calculated after Eq. (2)
$f_{\mathrm{res}}$	Hz	*var*	Measured by ARMS
Q	-	*var*	Measured by ARMS
$V_{R}L_{R}^{-1}$	m^2^	12,732.3954	Calculated
$V_{\mathrm{mol}}$	m^3^mol^−1^	*var*	Calculated for each measurement
$N_{A}$	mol^−1^	6.02E+23	Avogadro's constant
$\sigma_{i}\left({\overset{\sim }{\nu }}_{\mathrm{Ph}}\right)$	m^2^	5.06E-24	Adapted from Gierczak et al. [62]
$P_{0}({\overset{\sim }{\nu }}_{\mathrm{Ph}})$	W	*var*	Measured
$\epsilon_{\mathrm{relax}}$	-	1	n.d.

## 4 Experiments and results

### 4.1 Sensor calibration

The linearity of the LED-PAS acetone sensor was verified setting five acetone concentrations namely (1000, 500, 200, 100, 50) ppbV (MFC-mode) with a flow rate of 500 ml/min. Reference values were provided by the PTR-TOF-MS. Finally, the background signal without acetone was measured using SA to determine the noise level for LoD calculation. However, the measurements obtained from the PTR-TOF-MS consistently appeared significantly lower than the set MFC concentrations ($N_{\mathrm{Bottle}}\approx N_{\mathrm{PTR}}∙1.64$) for all measurements. Potential ideas on this discrepancy between the gas concentrations specified by the manufacturer and measured by the reference are:

• Incorrect gas tank concentration: The calibration gas was acquired approximately 1.5 years before the measurements were carried out. According to the manufacturer, the concentration of the bottle is guaranteed for only 6 months.
• Humid calibration-based quantification of PTR-TOF-MS data: Typically, in breath measurements with a PTR-TOF-MS, the sample is directly drawn in side-stream mode from a sterile breathing mouthpiece. During this process, the samples remain fully saturated with water, i.e. a relative humidity of 100%. The conversion of detected counts per second (cps) to ppbV is designed for water saturation. In the described experiments, where the relative humidity remains <100%, absolute concentrations may differ minimally by 2–5%.
• Loss of analyte within the system: Acetone is a polar VOC dissolving in water. Thus, the effective analyte concentration may be decreased due to humid surfaces of the tubing parts.
• A leak in the T-piece connection to the PTR. This is less likely since concentration measurements were performed under both positive pressure (MFC-Mode) and negative pressure (Bag mode). If the gas line were leaking, the deviation from the bottle concentration would not be identical in both measurements.

**Fig. 6:**
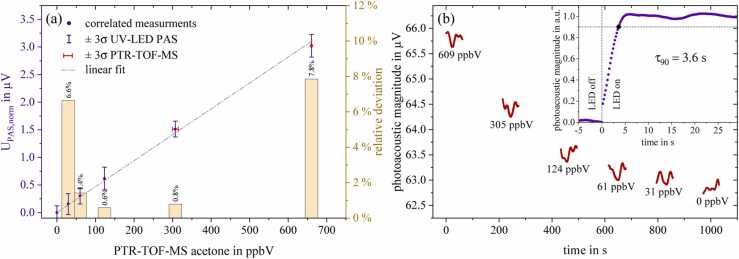
(a) Sensor calibration additionally illustrating the deviation from the PTR-TOF-MS reference and (b) sensor response with response time evaluation of PA signal generation.

Nevertheless, given that the PTR-TOF-MS is a reliably calibrated reference instrument, it is assumed that its concentration readings are accurate. This is further supported by the reproducible sensitivity of the PAS sensor, which is also consistent with previous laboratory measurements and different gas piping. As described, the calibration measurements with acetone were likewise pressure and offset-compensated ((3), (4)) and subsequently calibrated using PTR-TOF-MS measurement values (see Fig. 6a). The calibration curve was established through a linear fit of the calibration points and background signal measurement. Hence, the relative deviation at zero set acetone is 0%. The deviation of the calibration measurements to the PTR-TOF-MS values can be read from the bars referring to the right y-axis. This calibration resulted in a refinement factor of 4.834, which was applied for the conversion of all real breath measurements in this study. Based on this calibration, the sensitivity of the UV-PAS acetone sensor is 4.92 µV/ppmV (R^2^=0.99992), the 3σ noise level is 40.9 nV yielding an LoD(3σ) of 8.3 ppbV. From this, the normalized noise equivalent absorption coefficient (NNEA) was calculated to 1.4E-9 Wcm^−1^Hz^−0,5^ after Eq. (6) to allow comparison with other optical systems and thus facilitate the evaluation of sensor performance.

(6) $$\mathrm{NNEA}=\frac{\mathrm{LoD}\;N_{A}\sigma_{i}\left({\overset{∼}{\nu }}_{\mathrm{Ph}}\right){P_{0}\left({\overset{∼}{\nu }}_{\mathrm{Ph}}\right)}_{\mathrm{cor}}\;}{\mathrm{SNR}\sqrt{∆f}V_{\mathrm{mol}}}$$

Here, LoD represents the detection limit, which is determined as SNRtimes the standard deviation. The equivalent noise bandwidth ∆f of the LIA has been set to 0.0556 Hz. SNR is the multiple of standard deviation used to calculate the LoD i.e., 3. To meet the high divergence of the LED, a real optical power of 100 mW, which is approximately ${\text{C}}_{\text{cor}}$ times the measured optical power, was assumed for ${P_{0}({\tilde{\nu }}_{\mathrm{Ph}})}_{\mathrm{cor}}\;$for NNEA calculation.

Fig. 6(b) supplementary shows the response of the photoacoustic sensor for the five acetone concentrations that have been set for calibration. The photoacoustic raw-data signals while adjusting the acetone concentration have not been plotted for improved data visibility. However, to emphasize the piping of the gas mixing system to be responsible for the inertia of the PA signal to settle, the picture-in-picture illustrates the response behaviour of PA signal generation. For this purpose, a constant analyte concentration has been set and the LED light source was shaded. Removing the shutter at t = 0 s that represents a quasi-Heaviside step function yielded a response time of τ_90_ = 3.6 s at a lock-in time constant of 2 s.

### 4.2 UV-LED-PAS breath analysis

**Fig. 7:**
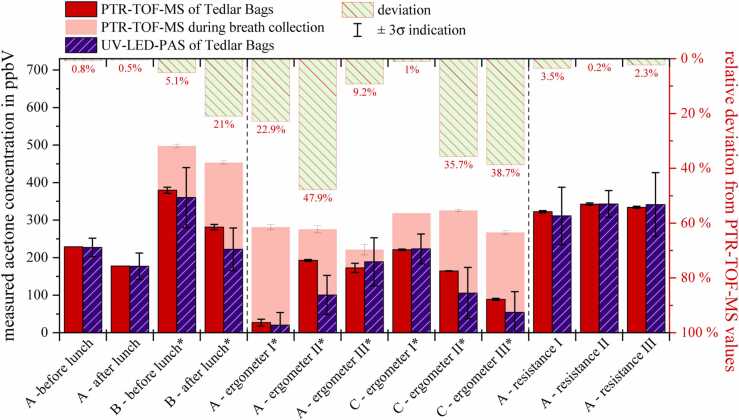
Acetone content of real breath samples measured by UV-LED-PAS (blue) and PTR-TOF-MS (red). The relative deviation of the UV-LED-PAS from the PTR-TOF-MS measurements are visualized by the upper x-axis bars. Breath resolved analysis done via PTR-TOF-MS during probe collection (light red bars) were only available where marked with a (*).

To the best of our knowledge, this section for the first time presents results obtained from actual breath samples performed with a UV-LED based photoacoustic acetone sensor. Fig. 7 illustrates a bar chart comparing PTR-TOF-MS data (in red) with UV-LED-PAS concentrations (in blue) along with error bars. Where available, the PTR-TOF-MS measurements during breath collection were also visualized by the light red bars. The percentage deviation of the PAS measurements is indicated by the bars on the upper x-axis with their level of deviation corresponding to the right y-axis. Breath samples were collected under three different scenarios, denoted by vertical dashed lines: before and after lunch, at three different exercise levels on an ergometer, and with sample collection after breath holding for a few seconds using an increased respiratory resistance (straw). The breath samples were collected from three volunteer participants, labeled as A, B and C.

In the breath samples collected in TBs before and after lunch, the UV-LED-PAS and PTR-TOF-MS measurements showed strong agreement. As expected, post-meal breath acetone concentrations decreased due to postprandial metabolic adaptions [48]. These indicate the real-time effects due to changes in energy metabolism on exhaled acetone profiles. This trend is also in accordance with the original breath-resolved analysis done via PTR-TOF-MS during probe sampling.

During the ergometer measurements, breath samples were initially collected during low physical exertion (I), followed by sample collection during high-intensity exercise (II). Phase III denotes the recovery phase, with no further physical effort. These measurements exhibit the most significant discrepancies between the UV-LED-PAS and PTR-TOF-MS systems, especially evident in cases (A – ergometer II) and (C – ergometer II). Potential explanations for these discrepancies are:

• If anything changes in the VOC profiles during breath measurements due to immediate physiological (ventilatory and/or hemodynamic) effects, it could be recognized immediately in the breath-resolved PTR-TOF-MS analysis (time resolution: 200 ms). Here, we overlayed several breath cycles into the bags and therefore, observed relatively higher variations (e.g., in dynamic situations such as ergometer setup) in the sensor system.
• As the sensor performance of the UV-LED-PAS system declines at lower analyte concentrations, an increased deviation from the PTR signal is anticipated.

Additionally, the trends observed across the three phases in the two participants are divergent. While in the case of participant A, the PTR-TOF-MS acetone concentration increases between phases I and II and remains at that level during phase III, it consistently decreases in terms of participant C. Schubert et al. observed a pattern in acetone concentration during the increase of physical workload, noting an increase up to the anaerobic threshold, a subsequent plateau, and a decline after surpassing this limit [17]. This was further confirmed within the recent study by Pugliese et al. [63]. Breath-resolved analysis via PTR-TOF-MS has depicted such increase in acetone in both A and C. During the Bag measurements, such progression is identified by UV-LED-PAS and PTR-TOF-MS within the collected breath samples from participant A. The discrepancy of C may indicate incorrect collection of the end-tidal phase. The overall marked differences in breaths vs. bags acetone concentrations further indicate an inaccurate CO_2_-trigger under relatively high respiratory rates (due to exercise) which may lead to mixed-expired sampling. Besides, as indicated above diffusion and/or aqueous binding of acetone may additionally contribute to such discrepancies. However, given that our study primarily focuses on the comparison of both analytical techniques, the emphasis lies on the sensor performance. The determined concentration values are consistent, except for point (A - ergometer II), falling within the range of three times the standard deviation.

The acetone breath analysis consistently demonstrated strong agreement within the measurements where samples were collected after breath holding for a few seconds through an increased flow resistance by a straw. This led to increased acetone within the exhaled breath. Both PTR-TOF-MS and the UV-LED-PAS sensor precisely quantified this heightened acetone concentration.

## 5 Conclusion

In this work, we introduced an innovative approach for breath acetone analysis utilizing inexpensive UV-LED photoacoustic spectroscopy. A central element is the development of a special breath sampling system that is customised for the collection of end-tidal breath. End-tidal breath represents the systemic concentrations of VOCs and therefore, provides clinically relevant information about various *in vivo* processes within our body. The Breath samples were collected in Tedlar bags over several minutes and then drawn through the UV-LED-PAS sensor using negative pressure. To ensure the reliability and accuracy of our system a PTR-TOF-MS served as a reference device. This instrument for real-time analysis was serially integrated into the gas stream in side-stream mode to allow precise calibration measurements and verification. The calibration yielded an LoD(3σ) of 8.3 ppbV and an NNEA of 1.4E-9 Wcm^−1^Hz^−0.5^. During the analysis of collected breath samples, the results occasionally deviated with the physiological expectations and/or with the breath-resolved observations via PTR-TOF-MS. Nevertheless, in 12 of 13 measurements, they fell within the 3σ error margin of the UV-LED-PAS measurements.

However, this demonstrator setup is not yet suitable for widespread bedside use. Key challenges that need to be addressed in further developments towards a point-of-care device are the breath sampling process and UV-LED-PAS sensor optimization. The goal is to determine the acetone content in exhaled breath with just a few or even one single breath cycle. Although collecting samples in Tedlar bags helps to simplify the measurement process, it may lead to a diffusion driven dilution of polar compounds and/or dissolving of the acetone in water condensate. Therefore, the future development should address both optimization of the existing CO_2_-trigger under high respiratory rate and modification of this sensor system to allow for direct analysis – without any sample storage. Furthermore, the high photoacoustic background signal and low SBSR present difficulties regarding repeatability and reproducibility. Additionally, the background signal level often fluctuated more than the amplitude induced by the sample from one measurement to another. Attempts were made to compensate for this interference by zeroing the background signal shortly after every breath measurement. Nevertheless, this work provides valuable insights into the development of low-cost optical sensing alternatives to expensive gold standard devices. By demonstrating that a trace (ppbV-level) detection limit and reliable in-field measurements could be achieved even with low-cost components, the goal of making non-invasive physio-metabolic monitoring accessible to a broad population becomes more attainable.

## Funding

This work has received essential financial support through the BreathSens project, funded by the German Ministry of Education and Research (10.13039/501100002347BMBF) under grant code 13GW0325C, and the PreSEDA project, funded by the German Federal Ministry for Economics and Climate Action (BMWK) under grant code 03EN2028A. Additional funding was provided by the BayWISS-Health network, supported by the Bavarian Ministry of Research and Arts. Furthermore, one of the authors is the recipient of a PhD scholarship from the Studienstiftung des Deutschen Volkes and the Marianne-Plehn-Programm of the Elitenetzwerk Bayern, funded by the Bavarian Ministry of Research and Arts.

## CRediT authorship contribution statement

**Jonas Pangerl:** Writing – review & editing, Writing – original draft, Visualization, Validation, Software, Methodology, Investigation, Funding acquisition, Formal analysis, Data curation, Conceptualization. **Pritam Sukul:** Writing – review & editing, Methodology, Investigation, Data curation, Conceptualization. **Thomas Rück:** Writing – review & editing, Validation, Methodology, Conceptualization. **Patricia Fuchs:** Writing – review & editing, Validation, Data curation, Conceptualization. **Stefan Weigl:** Writing – review & editing, Software, Methodology, Funding acquisition, Conceptualization. **Wolfram Miekisch:** Writing – review & editing, Validation, Supervision, Conceptualization. **Rudolf Bierl:** Writing – review & editing, Supervision, Project administration, Funding acquisition. **Frank-Michael Matysik:** Writing – review & editing, Validation, Supervision, Methodology, Conceptualization.

## Declaration of Competing Interest

The authors declare that they have no known competing financial interests or personal relationships that could have appeared to influence the work reported in this paper

## Data Availability

Data will be made available on request.

## Footnotes

1 Due to the low SBSR and a fluctuating background signal in both magnitude and phase, a phase-correct offset correction would increase the error in the determined acetone.
